# Rabbits Divergently Selected for Total Body Fat Content: Changes in Proximate Composition and Fatty Acids of Different Meat Portions

**DOI:** 10.3390/ani12182396

**Published:** 2022-09-13

**Authors:** Marco Cullere, Zsolt Szendrő, Zsolt Matics, Zsolt Gerencsér, Rozália Kasza, Tamás Donkó, Antonella Dalle Zotte

**Affiliations:** 1 Department of Animal Medicine, Production and Health, University of Padova, Agripolis, Viale dell’Università 16, Legnaro, 35020 Padova, Italy; 2 Department of Physiology and Animal Health, Hungarian University of Agriculture and Life Sciences, Kaposvár Campus, Guba Sándor u. 40, H-7400 Kaposvár, Hungary; 3 Department of Animal Science, Hungarian University of Agriculture and Life Sciences, Kaposvár Campus, Guba Sándor u. 40, H-7400 Kaposvár, Hungary; 4 Medicopus Nonprofit Ltd., Guba Sándor u. 40, H-7400 Kaposvár, Hungary

**Keywords:** divergent selection, rabbit, computed tomography, meat quality, health, consumer

## Abstract

**Simple Summary:**

The purpose of the investigation was to determine the effect of the selection for the total fat content of the rabbit body on the fat content and fatty acid composition of different meat cuts. The higher the fat content of the given meat, the more effective the selection was. Authors found that the meat of fatter rabbits is healthier, which can be used in the production of baby food.

**Abstract:**

The present research studied the potential of a four-generation divergent selection (Pannon maternal rabbit lines) based on the total body fat content to create two rabbit lines with different meat attributes: a Fat line to deliver greater amounts of healthy fatty acids for newborns and infants, and a Lean line intended to provide lean meat for everyday consumption. Selection was based on the fat index calculated in 10-week-old live rabbits by means of computed tomography (CT). For each generation, 60 rabbits/line were fed *ad libitum* with commercial pellet from weaning (5 weeks) to slaughter (11 weeks). A total of 15 rabbits/line were randomly selected for meat quality evaluations: the *longissimus thoracis et lumborum* muscles (LTLs), hind legs (HLs), forelegs (FLs) and abdominal wall (AW) were analyzed for their proximate composition and fatty acid (FA) profile. FA contents were also calculated. Results highlighted that it was possible to obtain leaner meat for everyday consumption in most meat portions starting from generation 4 (7.93 vs. 11.9, 5.10 vs. 5.98 and 7.26 vs. 10.9 g of lipids/100 g of meat in Lean and Fat groups for the FLs, HLs and AW, respectively). The sole exception was the LTLs, which were not affected by the divergent selection. The total PUFA amount increased in FL and AW (*p* < 0.05) portions of the Fat line only, attributable to a greater *n*-3 amount (151 vs. 216 and 73 vs. 143 mg/100 g of meat in Lean and Fat groups for the FLs and AW, respectively).

## 1. Introduction

One of the main selection criteria in rabbits is the prolificacy of maternal lines, expressed as litter size at weaning, because of its great economic relevance in intensive rabbit production [1]. Key components for achieving this goal are the number of kits born alive, their survival and doe’s milk production. However, the productive life of a rabbit doe is challenging (insemination performed about 12 days’ *postpartum*) as it requires a high-energy intake to have enough energy for growth (primiparous does) or maintenance (multiparous does), growth of fetuses and milk production. Due to the intense physiological stress, at the end of pregnancy and at the peak of lactation, the energy balance of rabbit does is often negative, leading to intense body fat reserve mobilization to cope with energy requests [2]. This can cause different reproductive problems and a suboptimal body condition, which are responsible for a high rate of culling and annual replacement [3].

Selection schemes for terminal lines dedicated to meat production are focused, instead, on growth rate, feed efficiency and meat yield [4,5]. Although farmers are still paid based on the weight of slaughtered rabbits or carcasses, meat quality is still not considered in most meat markets, including rabbit. The latter is, however, a condition that could and should gradually change. In fact, to stimulate consumers’ expectations on rabbit meat, and thus, promote it as a future choice, the improvement of the intrinsic quality of the product as well as the creation of value-added products are two possible strategies to relaunch a meat sector that, in Western countries, has been shrinking in the last decade [6]. An intrinsic attribute that is positively associated with modern consumers’ choices is product healthiness, and the amount of meat fat is surely associated with this as well. Faster-growing paternal lines have lower fat content than smaller-bodied lines [7]. Therefore, consumers’ expectations for less fatty meat are met with the terminal lines of hybrids. The rabbit carcass has an average fat content of about 6%, with significant variability depending on the considered meat cut [8]. On the one hand, consumers are requesting meat and meat products with a low fat content, but it is also true that rabbit meat has a balanced fatty acid profile in terms of SFAs, MUFAs and PUFAs, which can be further enhanced for consumers’ health [6].

The idea behind the present research is to exploit computed tomography (CT) to perform a selection of different populations of rabbits in function of the desired productive/commercial target [9]. The genetic selection of rabbits by exploiting CT has the advantage of allowing an effective evaluation of the body composition in vivo, avoiding animal slaughtering. At Kaposvár University (Hungary), CT selection has been operated since 1990, with satisfactory results for the selection of carcass traits [10].

The experiment of Kasza et al. [11] proved that it is possible to successfully select for increasing and decreasing fat depots, thus offering the opportunity (1) to provide energy reserves that rabbit does can mobilize when needed, and (2) to increase productive efficiency and reduce slaughter waste in terminal lines. The purpose of the present experiment is to investigate how the divergent selection for the total fat content of the body affects the fat content and fatty acid composition of different meat cuts.

## 2. Materials and Methods

### 2.1. Animals and Experimental Design

The experiment was conducted at the rabbitry of the Hungarian University of Agriculture and Life Sciences, Kaposvár Campus (Hungary). All rabbits were handled according to the principles stated in the European Directive 2010/63/EU regarding the protection of animals used for experimental and other scientific purposes [12], and according to the Hungarian legal requirements (32/1999./III. 31./and 178/2009./XII. 29./).

The Pannon Ka rabbit (selected on the number of kits born alive; [11]) was involved in the divergent selection program for total body fat content. Selection was based on the fat index calculated in 10-week-old live rabbits by means of CT. The rabbits with low fat indexes formed the Lean-selected line, whereas those with high fat indexes formed the Fat-selected line. The divergent selection was carried out for four generations: about 33–70% of CT-scanned females and 32–50% of CT-scanned males were selected as Fat or Lean breeding rabbits. Fat and Lean rabbit does were inseminated with the semen from selected bucks of the same line. The detailed description of the divergent selection protocol and further details of the fat index calculation and selection scheme procedures is provided in the work by Kasza et al. [13].

For each generation, a total of 60 rabbits/line were housed in wire-mesh cages (62.5 cm × 32 cm × 30 cm; 3 rabbits/cage; 16 rabbits/m^2^) and ad libitum fed with commercial pellet from weaning (5 weeks) to slaughter (11 weeks). Two commercial diets were used: one medicated diet was offered to rabbits between 5 and 9 weeks of age (15.7% crude protein, 2.4% ether extract and 9.9 MJ of digestible energy/kg feed), and one non-medicated diet during the last two weeks of farming (16.3% crude protein, 3.8% ether extract and 10.6 MJ of digestible energy/kg feed). Drinking water was always available from nipple drinkers. The daily lighting period was 16 h and the average environmental temperature was in the range 15–18 °C, with the exception of generation 2 which was conducted in summer (20–28 °C).

At 11 weeks of age, rabbits were transported to the commercial slaughterhouse located 200 km away from the rabbit farm, and slaughtered after electric stunning. Carcasses were then dissected following the guidelines of the World Rabbit Science Association [14]. Prior to slaughter, the rabbits were fasted for 6 h (including transport).

A total of 15 male rabbits/line were randomly selected (1 rabbit/cage) for meat quality evaluations. From each rabbit, the *longissimus thoracis et lumborum* muscles (LTLs), hind legs (HLs), forelegs (FLs), and abdominal wall (AW) were excised, individually vacuum-packaged in polyethylene bags, frozen (−40 °C) and transported under crushed ice to the Department of Animal Medicine, Production and Health (MAPS) of the University of Padova, Italy for meat quality evaluations.

### 2.2. Sample Preparation and Proximate Composition

After one month of frozen storage (−40 °C), rabbit samples were thawed (12 h at +4 °C), and ground with a Retsch (Retsch Italia, Verder Scientific S.r.l., Torre Boldone, BG, Italy) Grindomix GM 200 (7000 g for 10 s). Once ground, meat samples were frozen at −40 °C, freeze-dried and ground again (7000 g for 5 s) to obtain a fine powder, which was used to determine the proximate composition and FA profile. The proximate composition of meat samples was analyzed in accordance with the Association of Official Analytical Chemists [15] methods: moisture (method no. 934.01), crude protein (method no. 2001.11) and ash (method no. 967.05). Ether extract (EE) was analyzed after acid hydrolysis [16].

### 2.3. Fatty Acid Profile and Contents

The lipid extraction was performed using modified accelerated solvent extraction (M-ASE), exploiting a binary solvent mixture of chloroform/methanol 1:2 according to the method described by Lee et al. [17]. The fat content of samples was determined gravimetrically after vacuum evaporation under nitrogen stream. Afterwards, samples were transmethylated using a 4% H_2_SO_4_ methanolic solution to determine FA methyl esters (FAMEs). In each sample, a biphasic separation was achieved by adding 0.5 mL of distilled water and 1.5 mL of n-heptane. FAMEs were quantified via gas chromatography (Shimadzu GC17A), equipped with an Omegawax (Sigma-Aldrich Co. LLC, Saint Louis, MO, USA) 250 column (30 m × 0.25 μm × 0.25 μm) and flame ionization detector. Helium was used as the carrier gas at a constant flow of 0.8 mL/min. The injector and detector temperatures were 260 °C. Commercially available FAME mixtures (37-Component FAME Mix; Supelco Inc., Bellefonte, PA, USA) were used for identification. Results were expressed as the % of total detected FAMEs. Finally, the chromatographic peak area, the internal standard (C19:0) method and the total lipid content of the sample were exploited for the quantitative determination of FAs (w/w). The lipid peroxidability (PI) indexes of the four meat portions were calculated as reported by Dalle Zotte et al. [18].

### 2.4. Statistical Analysis

Results about the proximate composition and fatty acid profile of the different meat portions considered in the present study were subjected to a two-way ANOVA with the selection group (Fat and Lean), generation (1st, 2nd, 3rd and 4th for proximate composition; 3rd and 4th for fatty acid profile) and their interaction as fixed effects following the GLM (general linear model) procedures of the SAS 9.1.3 statistical analysis software for Windows [19]. Least square means were obtained, and a post hoc pairwise comparison was performed using the Bonferroni correction. The significance was considered at a 5% confidence level. When the interaction was not significant, only the single effect group and generation were presented.

## 3. Results

### 3.1. Proximate Composition

Results presented in Table 1a,b show the effect of the rabbit line (Lean and Fat), generation of divergent selection (1–4) and their interaction on the proximate composition of four different rabbit meat portions: LTLs, FLs, HLs, and AW. At generation 1, the four meat portions showed the same proximate composition in Lean and Fat rabbits (*p* > 0.05). Then, generation after generation, the selection effort effectively created two different populations of rabbits, showing different meat quality characteristics. The magnitude of the asymmetric change depended on the considered meat portion: the fatter the meat portion, the higher the efficacy of the selection program. Specifically, the comparison of Lean and Fat groups within a generation for the LTL cut highlighted that the lipid content was similar (*p* > 0.05) in the two groups in all four generations. A comparable outcome was noticed for the HLs in the first two generations but, by generation 3 of divergent selection, the lipid content showed a tendency (*p* < 0.10) for a higher content in the Fat group compared to the Lean one. Such a tendency became a significant effect (*p* < 0.001) at generation 4, where the Fat group had a higher lipid content compared to the Lean one (5.98 vs. 5.10 g/100 g of meat for Fat and Lean groups, respectively).

For FLs and AW, two different rabbit groups in terms of meat proximate composition were highlighted already at generation 2 of divergent selection, and the difference was maintained also in the following generations (3 and 4). The lipid amount was, however, not the sole quality parameter that was affected by the divergent selection for total body fat content. The increase/decrease in lipids was accompanied by an opposite modification in the moisture content (*p* < 0.001), with protein remaining unaffected (*p* > 0.05). The only exception was the FLs at generation 4 of divergent selection: together with moisture and lipid contents, protein also differed in the Lean and Fat groups (16.2 vs. 15.2 g/100 g of meat for Lean and Fat groups, respectively; *p* < 0.01).

**Figure 1:**
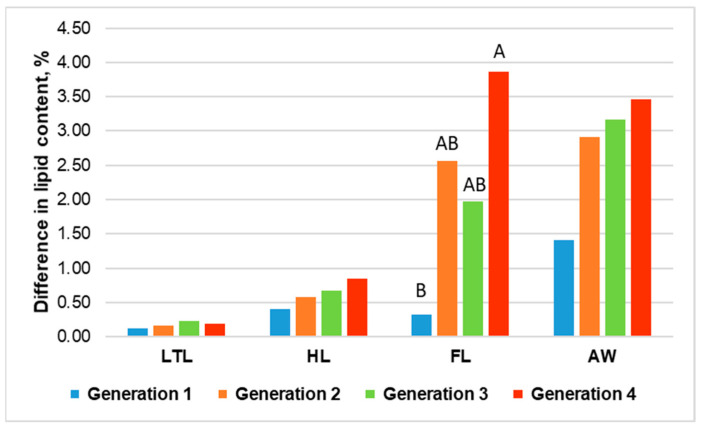
Differences between the lipid content of meat cuts (LTL: *longissimus thoracis et lumborum* muscle; HL: hind leg; FL: foreleg; AW: abdominal wall) of the Fat and Lean lines depending on the generation.

The generation effect highlighted significant differences (*p* < 0.05) for all meat cuts considered in the present study (Table 1a,b), which was attributable to the progressive and cumulative modifications in the proximate composition of each rabbit meat cut as a result of the divergent selection for each generation, independent of the selection group. Therefore, the strong generation effect indicates that the chemical composition of the rabbit meat cuts changed over the four generations, but a seasonal/group bias is masked within this effect. Differently, the real impact of the divergent selection in the four generations can be better appreciated by the difference between the lipid content of the Fat and Lean lines in the four meat portions presented in Figure 1.

The difference between the two lines in all meat cuts increased from generation to generation, with a magnitude related to the absolute lipid content of the meat cut. Here, the differences in the lipid content from generations 1 to 4 in the four meat cuts are reported: 0.12-0.16-0.23-0.19% for the LTLs, 0.41-0.58-0.67-0.85% for the HLs, 0.32-2.56-1.97-3.86% for the FLs, and 1.41-2.91-3.16-3.47% for the AW. However, a significant effect was observed for the different lipid content in generations 1 and 4 of the FLs only, whereas 2 and 3 were intermediate (*p* < 0.001). In the other meat cuts, no significant differences were observed as a result of the relatively limited sample size and data variability.

The interaction, Line x Generation, showed a significant effect only for moisture (*p* < 0.05), and lipid (*p* < 0.01) contents of the FLs: the moisture content and lipids increased and decreased, respectively, in Lean rabbits. In the Fat group, instead, the lipid content was stabilized by generation 3 of divergent selection, whereas the moisture content was similar in the four generations (*p* > 0.05).

### 3.2. Fatty Acids

To improve results readability, data presented in this section refer to the main FA of rabbit meat and those that have been significantly affected by the tested treatments. The complete FA profile of the considered meat portions is reported in the supplementary tables (Tables S1–S4). The divergent selection for total body fat content changed FA classes in the considered rabbit meat portions. Looking at the LTL FA profile (Table 2), in generation 3, MUFA was the only class which was already different in the two rabbit populations: 30.6 vs. 32.7% for Lean and Fat groups, respectively. Such a result was mainly attributable to C16:1 FA (1.73 vs. 2.81% for Lean and Fat groups, respectively; *p* < 0.001). One generation later, the selection was definitively more effective as it was highlighted by differences in MUFA (*p* < 0.001), PUFA (*p* < 0.01) and some SFA (C14:0, C16:0, C17:0 and C18:0) proportions. Regarding PUFAs, the shift regarded the *n*-6 series: it was lower in the Fat than in the Lean group (26.9 vs. 30.0% for Fat and Lean groups, respectively; *p* < 0.001), which also led to a lower, and thus better, *n*-6/*n*-3 ratio (15.1 vs. 20.1 for Fat and Lean groups, respectively; *p* = 0.001). Specifically, the main PUFAs which caused the above-mentioned shift were C20:4 *n*-6 (*p* < 0.01) and C20:3 *n*-6 (*p* < 0.05).

A similar outcome was observed for the hind leg FA (Table 3): in generation 3 of divergent selection, Lean and Fat rabbits showed substantially similar FA profiles (*p* > 0.05). Differently, in generation 4, significant modifications were detected and they followed the same trend highlighted for LTL FAs: MUFAs were higher in the Fat than in the Lean group (30.8 vs. 26.4% for Fat and Lean groups, respectively; *p* < 0.001), whereas PUFAs were lower in the Fat than in the Lean group (28.5 vs. 32.3% for Fat and Lean groups, respectively; *p* < 0.001). Again, this change was due to the FAs of the *n*-6 series, with linoleic (C18:2 *n*-6; *p* < 0.01) and arachidonic (C20:4 *n*-6; *p* < 0.01) acids being the main drivers. Additionally, for the hind leg FA profile, Fat rabbits showed a better *n*-6/*n*-3 ratio (12.6 vs. 15.0 for Fat and Lean groups, respectively; *p* < 0.001).

Fatty acids of the FLs (Table 4) and the AW (Table 5) exhibited results that overlapped with those described for the HLs. A difference was noticed for the overall PUFA series, which was similar (*p* > 0.05) in the two groups of rabbits for both generations 3 and 4 and both rabbit meat cuts. Despite this, the *n*-6 series was always lower in Fat than in Lean rabbits, leading to an improvement in the *n*-6/*n*-3 ratio and, consequently, meat healthiness: 11.5 vs. 13.4 for the forelegs (*p* < 0.01), 12.5 vs. 15.9 for the abdominal wall (*p* < 0.001).

As it was observed for the results of the proximate composition and FA profiles, while also considering the results of FA amounts, the magnitude of the selection effect followed a gradient related to the total lipid amount of each meat portion: the fatter the meat portion, the greater the effect of the selection: LTL < HL < FL < AW (Table 6). The considered meat portions of Lean and Fat rabbit lines showed different fatty acid contents in generations 3 and 4 of divergent selection. In generation 3, the selection effect was evident in all meat cuts, but acted differently depending on the portion: for LTLs and HLs, only the MUFA content was different in Lean and Fat rabbits (871 vs. 1063 mg/100 g of meat for Lean and Fat rabbits, respectively; *p* < 0.05). For FLs, again, the MUFA content differed (2625 vs. 3414 mg/100 g of meat for Lean and Fat rabbits, respectively; *p* < 0.01), as did the *n*-3 content (199 vs. 263 mg/100 g of meat for Lean and Fat rabbits, respectively; *p* < 0.05). For the AW, all FA classes were affected by selection: SFA (2613 vs. 3540 mg/100 g of meat for Lean and Fat rabbits, respectively; *p* < 0.05), MUFA (2746 vs. 3895 mg/100 g of meat for Lean and Fat rabbits, respectively; *p* < 0.001), PUFA (2914 vs. 3727 mg/100 g of meat for Lean and Fat rabbits, respectively; *p* < 0.05), *n*-6 (2696 vs. 3427 mg/100 g of meat for Lean and Fat rabbits, respectively; *p* < 0.05), and *n*-3 (218 vs. 300 mg/100 g of meat for Lean and Fat rabbits, respectively; *p* < 0.05) fractions.

In generation 4 of divergent selection, MUFAs were again the highly affected FA series, being different in Lean and Fat lines for all meat portions. Different from the results observed in generation 3, in generation 4 the selection seemed to produce a greater difference between the two lines. In fact, in the HLs, SFAs were also higher in Fat than in Lean rabbits (*p* < 0.001), whereas in the FL meat both SFAs (*p* < 0.001) and PUFAs (*p* < 0.05) were higher in Fat than Lean rabbits. The PUFA difference was attributable to the *n*-3 FA in the Fat line being twice the amount found in the Lean group (*p* < 0.05). Considering the AW, SFAs (*p* < 0.01), MUFAs (*p* < 0.001), and *n*-3 PUFAs (*p* < 0.05) were also higher in Fat than in Lean rabbits. However, different from what was observed in generation 3 of divergent selection, in generation 4, overall, PUFAs and the *n*-6 series were similar (*p* > 0.05) in Lean and Fat rabbits. Interestingly, the results highlighted also that the absolute amount of AW PUFAs were strongly reduced in generation 4 compared to the amount found in generation 3 of divergent selection. This result could be attributable to the different moisture (generation 3: 68.6 g of moisture/100 g of meat; generation 4: 74.0 g of moisture/100 g of meat) and lipid (generation 3: 12.1 g of lipids/100 g of meat; generation 4: 9.08 g of lipids/100 g of meat) contents of AW samples in generations 3 and 4 of divergent selection.

A further consideration that arises when observing the data in Table 2, Table 3, Table 4, Table 5 and Table 6 is that when a significant difference between Lean and Fat lines was depicted in both generations, the magnitude of the mean difference between the two rabbit populations (value in parenthesis) always increased from generation 3 to 4. The sole exception was the *n*-3 FA content of the FLs and AW, whose difference between the Lean and Fat groups did not increase in generation 4 compared to generation 3.

## 4. Discussion

In general, the results confirm that the effectiveness of divergent selection in developing Lean and Fat lines based on total body fat content [11] was also reflected in the lipid content of the studied meat cuts.

When selecting for total body fat content, different fat depots (visceral, subcutaneous, intermuscular and intramuscular) are involved. Intramuscular fat surely is of interest for consumers: nutritionally, since it provides FAs, and hedonically, because it affects the sensory attributes of the meat [20]. However, given that the total intramuscular fat content in a rabbit carcass is extremely low, roughly 1% [21], genetic selection for total body fat content is expected to exert only a marginal effect in this sense [4]. This was observed for the fat content of the LTL, which is the leanest meat cut of the rabbit carcass [8], and fat reduction cannot exceed a physiological threshold. The first results of the present experiment showed that after four generations of selection, the Lean and Fat lines diverged in the growth performance traits (feed conversion ratio), dressing-out percentage (different fore and hind part proportions) and carcass adiposity [11]. To the best of the authors’ knowledge, in the literature there are no other studies that tried to exploit the divergent selection for total body fat content to create two rabbit populations with different meat quality attributes by using CT. According to the results of Kasza et al. [11], the amount and proportion of fat depots around the kidneys and shoulder changed as a result of divergent selection for the total fat content of the body. The present experiment proved that this change can also be detected in the fat content of different meat cuts. Martínez-Álvaro et al. [22] conducted a divergent selection for intramuscular fat content over six generations, and fat deposits also changed. When divergent selection efforts were directed towards intramuscular fat content, seven generations [23] produced a 5% divergence in the mean value of the *longissimus thoracis et lumborum* muscle/generation and both rabbit lines followed a symmetrical trend. Considering other muscles, selection for intramuscular fat content generated correlated responses also in the *biceps femoris*, *supraspinatus* and *semimembranosus proprius* [22]. Recent findings have also highlighted that a ten-generation divergent selection for intramuscular fat content produced rabbit populations characterized by a high degree of genomic differentiation, with ten main genes involved and a large polygenic component [24].

The literature data shows that fat composition in rabbits is heritable to a certain extent, depending on the considered FA class: an experiment studying the responses to selection and genetic parameters of rabbits divergently selected for intramuscular fat found a high heritability for MUFAs (0.61) and PUFAs (0.41), whereas a low value (0.09) was observed for SFAs [23]. Furthermore, SFAs showed that they were positively correlated with intramuscular fat content but had wide confidence intervals, MUFAs were strongly and positively correlated, whereas PUFAs highlighted a strong but negative correlation [22]. The results of the MUFA proportion from the present research, considering a divergent selection for total body fat content, are coherent with the above-mentioned findings, whereas the PUFA percentage showed a decrease with the selection for high total body fat content. However, the latter was observed only in the LTLs and the HLs, whereas a tendency to decrease was observed for the FLs and no change was observed in the AW. Conversely, the SFA proportion was not affected by the selection. Overall, a strong generation effect was observed also in FA amounts, which was expected due to the ongoing selection process.

When it comes to FA contents, however, different information could be deducted; this is extremely relevant from the nutritional point of view: rabbits of the Fat group did not show a substantial change in SFAs when considering the leanest meat cut (LTL), up to generation 4 of divergent selection. Differently, meat portions of Fat rabbits with a higher lipid amount (HLs, FLs, AW) displayed also a greater SFA amount compared to Lean ones, but only starting at generation 4. Dietary recommendations on fatty acids highlighted that, within a nutritionally adequate diet, SFA intake should be as low as possible: when establishing nutrient goals and recommendations, limiting their intake should be considered. Numerically speaking, however, no dietary reference values for total intake of SFAs has been established yet [25]. Fat rabbits exhibited an augmented MUFA content in all rabbit meat portions, but this does not represent a nutritional issue, since this group of FAs has no known specific role in preventing or promoting diet-related diseases and their intake can account for up to 20% of the dietary energy [26]. Regarding total PUFAs, their dietary intake increase is a shared goal of different nutritional guidelines [25,27]. Results of the present trial highlighted that rabbits of the Fat line are headed in that direction, with regard to the PUFAs of the *n*-3 series, for FL and AW meat portions, whereas LTL and HL *n*-3 contents in Lean and Fat lines remained similar. Interestingly, the results highlighted also that the absolute amount of AW PUFAs was strongly reduced at generation 4 compared to the amount found at generation 3 of divergent selection. This result could be attributable to the different moisture (generation 3: 68.6 g of moisture/100 g of meat; generation 4: 74.0 g of moisture/100 g of meat) and lipid (generation 3: 12.1 g of lipids/100 g of meat; generation 4: 9.08 g of lipids/100 g of meat) contents of AW samples in generations 3 and 4 of divergent selection.

In growing rabbits (11 weeks old), lipogenesis was reported to occur mainly in the liver (about 60%), whereas a 35% was attributable to the adipose tissue [28]. MUFAs are endogenously synthesized from SFAs by exploiting the enzyme Δ9-desaturase [29] and both SFAs and MUFAs are exploited as energy stock; this explains the finding that Fat rabbits often displayed greater amounts of these FA groups than Lean ones. The literature on PUFA metabolism showed that in fast-growing rabbits, the activity of Δ6-desaturase, a key enzyme for long-chain *n*-6 and *n*-3 production from the precursor linoleic and linolenic acids, is almost entirely oriented toward the desaturation of linoleic acid rather than linolenic acid [30]. However, in the present study, elongase and desaturase enzymes, yet not directly investigated, might have worked differently in the two rabbit lines since different *n*-3 PUFA amounts were observed in Lean and Fat rabbits. This might be linked to the different lipid amounts observed in the Fat and Lean lines. In fact, the production of *n*-3 long-chain PUFAs, essential for several physiological functions, needs a greater metabolic expenditure than *n*-6 PUFAs since more enzymatic steps (elongation, desaturation and β-oxidation into peroxisomes) are required [31]. A further possible explanation of our results could come from a research study focusing on the liver metabolism of two rabbit lines divergently selected for intramuscular fat content [32]: it was observed that the high line displayed a greater liver weight and lipogenic activities compared to the low line, and that both traits were positively correlated with the muscular deposition of fat.

## 5. Conclusions

The divergent selection for total body fat content by computed tomography was effective in reaching the objective to create two rabbit populations for different purposes. Results in the Lean group highlighted that, in certain meat cuts which are already lean, i.e., the *longissimus thoracis et lumborum* muscle, it is hard to achieve lower values since a physiological threshold cannot be crossed. Differently, selection acted to reduce the lipid content of the other meat portions starting at generation 4, therefore fulfilling the objective to produce lean meat for everyday consumption. The other target of the present research project was to obtain rabbit meat richer in healthy fatty acids for newborns and infants. Results in this case were partly satisfactory: total PUFAs increased in FL and AW meat portions only, whereas a greater *n*-3 amount was achieved. A better result in this sense can probably be achieved by feeding Fat rabbits with diets rich in PUFAs. The experimental results and experience can be used during the selection of other rabbit breeds, depending on the purpose of the selection.

## Figures and Tables

**Table 1:** (**a**) Proximate composition (g/100 g of meat) of the *longissimus thoracis et lumborum* (LTL) and forelegs (FLs) of Lean and Fat rabbits along four generations of divergent selection for total body fat content. (**b**) Proximate composition (g/100 g of meat) of the hind legs (HLs) and abdominal wall (AW) of Lean and Fat rabbits along four generations of divergent selection for total body fat content.

**(a) Meat Cut**			**LTL**				**FL**			
**Generation (Gen)**	**Line**	***n* Samples**	**Moisture**	**Protein**	**Lipids**	**Ash**	**Moisture**	**Protein**	**Lipids**	**Ash**
1	Lean	15	75.4	19.6	3.68	1.35	72.5 ^b,c^	16.7	9.58 ^c,d,e^	1.24
	Fat	15	75.0	19.9	3.79	1.32	72.2 ^b,c,d^	16.7	9.96 ^b,c,d,e^	1.14
	SE ^1^		0.16	0.15	0.09	0.01	0.35	0.11	0.42	0.03
	*p*-Line		ns ^2^	ns	ns	ns	ns	ns	ns	ns
2	Lean	15	75.9	19.1	3.61	1.42	72.9 ^a,b^	16.9	8.95 ^d,e^	1.24
	Fat	15	75.3	19.5	3.78	1.41	70.5 ^d,e^	16.6	11.7 ^a,b,c^	1.16
	SE		0.13	0.10	0.05	0.02	0.34	0.15	0.39	0.01
	*p*-Line		ns	ns	ns	ns	0.004	ns	0.003	ns
3	Lean	15	74.4	20.4	3.73	1.48	70.6 ^c,d,e^	17.7	10.3 ^a,b,c,d^	1.34
	Fat	15	74.2	20.3	3.96	1.57	68.8 ^d,e^	17.4	12.4 ^a^	1.34
	SE		0.12	0.10	0.06	0.06	0.36	0.13	0.43	0.03
	*p*-Line		ns	ns	ns	ns	ns	ns	ns	ns
4	Lean	15	75.2	19.4	3.96	1.45	74.6 ^a^	16.2	7.93 ^e^	1.19
	Fat	15	75.2	19.2	4.18	1.42	71.7 ^b,c,d^	15.2	11.9 ^a,b^	1.14
	SE		0.10	0.08	0.05	0.02	0.35	0.16	0.43	0.02
	*p*-Line		1.000	1.000	0.683	1.000	<0.0001	0.004	<0.0001	0.946
	*p*-Gen		<0.0001	<0.0001	<0.0001	0.0024	<0.0001	<0.0001	0.006	<0.0001
	*p*-Line x Gen		ns	ns	ns	ns	0.015	ns	0.004	ns
	SE		0.68	0.60	0.35	0.19	1.63	0.72	1.89	0.13
**(b) Meat Cut**			**HL**				**AW**			
**Generation (Gen)**	**Line**	***n* Samples**	**Moisture**	**Protein**	**Lipids**	**Ash**	**Moisture**	**Protein**	**Lipids**	**Ash**
1	Lean	15	75.1	18.6	5.01	1.37	70.4	19.0	9.46	1.2
	Fat	15	74.8	18.4	5.45	1.36	68.9	18.7	11.3	1.17
	SE		0.16	0.17	0.18	0.03	0.52	0.14	0.60	0.01
	*p*-Line		ns	ns	ns	ns	ns	ns	ns	ns
2	Lean	15	75.2	18.3	5.20	1.28	72.2	17.9	8.75	1.20
	Fat	15	74.4	18.5	5.85	1.26	69.7	17.3	11.8	1.13
	SE		0.14	0.06	0.11	0.01	0.41	0.21	0.50	0.03
	*p*-Line		0.038	ns	ns	ns	0.045	ns	0.024	ns
3	Lean	15	74.1	19.0	5.52	1.44	70.0	18.4	10.4	1.29
	Fat	15	73.3	19.0	6.27	1.41	67.2	17.8	13.8	1.26
	SE		0.13	0.06	0.14	0.02	0.47	0.17	0.56	0.02
	*p*-Line		ns	ns	ns	ns	0.013	ns	0.008	ns
4	Lean	15	76.2	17.4	5.10	1.33	75.6	16.1	7.26	1.13
	Fat	15	75.0	17.6	5.98	1.41	72.3	15.7	10.9	1.12
	SE		0.17	0.11	0.13	0.02	0.41	0.15	0.45	0.01
	*p*-Line		<0.001	ns	0.017	ns	0.002	ns	0.003	ns
	*p*-Gen		<0.0001	<0.0001	0.005	<0.0001	<0.0001	<0.0001	<0.0001	<0.0001
	*p*-Line x Gen		ns	ns	ns	ns	ns	ns	ns	ns
	SE		0.73	0.61	0.70	0.11	2.16	0.91	2.51	0.11

^1^ SE: standard error; ^2^ not significant; ^a–e^ means in the same column with different superscript letters significantly differ (*p* < 0.05).

**Table 2:** Fatty acid profile (% of total FAMEs) of the *longissimus thoracis et lumborum* of Lean and Fat rabbits in third and fourth generations of divergent selection for total body fat content.

Generation (Gen)	3				4				*p*-Gen	
Line	Lean	Fat	SE ^1^	*p*-Line	Lean	Fat	SE	*p*-Line		SE
Fatty Acid Profile	% Fatty Acid				% Fatty Acid					
*n*. samples	15	15			15	15				
C14:0	1.52	1.83	0.07	ns ^2^	1.09	1.78	0.09	<0.001	0.016	0.07
C16:0	21.3	21.6	0.30	ns	22.8	24.1	0.20	0.040	<0.001	0.24
C17:0	0.64	0.56	0.01	ns	0.75	0.59	0.03	0.006	0.050	0.02
C18:0	5.98	5.44	0.11	ns	9.51	8.29	0.20	<0.001	<0.001	0.14
SFAs ^3^	30.1	30.6	0.35	ns	35.5	35.9	0.20	ns	<0.001	0.29
C16:1	1.73	2.81	0.17	<0.001 (1.08 ^8^)	0.83	2.26	0.17	<0.001 (1.43)	<0.001	0.01
C18:1 *n*-9	26.7	28.1	0.43	ns	20.5	24.1	0.49	<0.001	<0.001	0.39
C22:1 *n*-9	0.03	0.06	0.02	ns	0.34	0.20	0.03	0.030	<0.001	0.03
MUFAs ^4^	30.0	32.7	0.54	0.018 (2.70)	23.9	28.8	0.61	<0.001 (4.90)	<0.001	0.45
C18:2 *n*-6	23.6	23.0	0.24	0.677	23.8	22.8	0.32	0.267	<0.001	0.28
C18:3 *n*-3	1.57	1.69	0.04	ns	1.10	1.47	0.08	0.009	<0.001	0.06
C20:3 *n*-6	0.16	0.13	0.03	ns	0.48	0.32	0.03	0.044	<0.001	0.03
C20:4 *n*-6	2.57	2.06	0.13	ns	5.31	3.37	0.31	<0.001	<0.001	0.20
C20:5 *n*-3	0.03	0.03	0.01	ns	0.22	0.14	0.02	0.007	<0.001	0.01
PUFAs ^5^	28.3	27.1	0.34	ns	31.6	28.8	0.43	0.001	<0.001	0.34
UFAs ^6^/SFAs	1.91	1.95	0.02	ns	1.57	1.60	0.02	ns	<0.001	0.02
*n*-6	26.6	25.4	0.34	ns	30.0	26.9	0.44	<0.001	<0.001	0.33
*n*-3	1.65	1.73	0.03	ns	1.59	1.87	0.08	ns	ns	0.06
*n*-6/*n*-3	16.2	14.8	0.30	ns	20.1	15.1	0.91	0.001	0.015	0.59
PI ^7^	38.7	36.1	0.68	ns	51.5	42.6	1.29	<0.001	<0.001	0.85
Identified, %	88.9	90.5			91.0	93.5				

^1^ SE: standard error; ^2^ not significant; ^3^ SFAs: saturated fatty acids; ^4^ MUFAs: monounsaturated fatty acids; ^5^ PUFAs: polyunsaturated fatty acids; ^6^ UFAs: unsaturated fatty acids; ^7^ peroxidability index: (% monoenoic × 0.025) + (% dienoic × 1) + (% trienoic × 2) + (% tetraenoic × 4) + (% pentaenoic × 6) + (% hexaenoic × 8); ^8^ difference between means.

**Table 3:** Fatty acid profile (% of total FAMEs) of the hind legs of Lean and Fat rabbits in third and fourth generations of divergent selection for total body fat content.

Generation (Gen)	3				4				*p*-Gen	
Line	Lean	Fat	SE ^1^	*p*-Line	Lean	Fat	SE	*p*-Line		SE
Fatty Acid Profile	% Fatty Acid				% Fatty Acid					
*n*. samples	15	15			15	15				
C14:0	1.90	2.06	0.06	ns ^2^	1.65	2.11	0.07	0.001	ns	0.06
C16:0	20.4	21.2	0.33	ns	23.1	24.5	0.26	ns	<0.001	0.29
C18:0	5.87	5.56	0.11	ns	8.47	7.59	0.14	0.001	<0.001	0.11
SFAs ^3^	30.5	31.0	0.40	ns	35.1	35.9	0.24	ns	<0.001	0.33
C16:1	1.65	2.45	0.13	0.003 (0.80 ^8^)	1.29	2.69	0.17	<0.001 (1.40)	ns	0.11
C18:1 *n*-9	29.9	30.2	0.20	ns	22.9	25.9	0.40	<0.001	<0.001	0.26
MUFAs ^4^	33.1	34.5	0.30	ns	26.4	30.8	0.56	<0.001	<0.001	0.33
C18:2 *n*-6	27.0	26.0	0.34	ns	26.7	23.8	0.43	0.001	0.015	0.34
C18:3 *n*-3	2.07	2.18	0.05	ns	1.71	1.71	0.08	ns	<0.001	0.06
C20:3 *n*-3	0.00	0.00	0.00	ns	0.19	0.20	0.03	ns	<0.001	0.03
C20:4 *n*-6	1.23	1.15	0.06	ns	2.74	1.94	0.18	0.009	<0.001	0.12
C22:2 *n*-6	0.00	0.00	0.00	ns	0.14	0.10	0.02	ns	<0.001	0.02
PUFAs ^5^	30.9	30.0	0.40	ns	32.3	28.5	0.53	<0.001	ns	0.40
UFAs ^6^/SFAs	2.11	2.09	0.03	ns	1.66	1.65	0.02	ns	<0.001	0.03
*n*-6	28.7	27.7	0.38	ns	30.3	26.7	0.47	<0.001	ns	0.36
*n*-3	2.15	2.22	0.04	ns	2.04	2.13	0.04	ns	ns	0.04
*n*-6/*n*-3	13.6	12.5	0.24	ns	15.0	12.6	0.36	<0.001	0.040	0.26
PI ^7^	38.0	36.9	0.55	ns	44.4	38.0	0.94	<0.001	<0.001	0.65
Identified, %	94.5	95.4			94.1	95.2				

^1^ SE: standard error; ^2^ not significant; ^3^ SFAs: saturated fatty acids; ^4^ MUFAs: monounsaturated fatty acids; ^5^ PUFAs: polyunsaturated fatty acids; ^6^ UFAs: unsaturated fatty acids; ^7^ peroxidability index: (% monoenoic × 0.025) + (% dienoic × 1) + (% trienoic × 2) + (% tetraenoic × 4) + (% pentaenoic × 6) + (% hexaenoic × 8); ^8^ difference between means.

**Table 4:** Fatty acid profile (% of total FAMEs) of the forelegs of Lean and Fat rabbits in third and fourth generations of divergent selection for total body fat content.

Generation (Gen)	3				4				*p*-Gen	
Line	Lean	Fat	SE ^1^	*p*-Line	Lean	Fat	SE	*p*-Line		SE
Fatty Acid Profile	% Fatty Acid				% Fatty Acid					
*n*. samples	15	15			15	15				
C12:0	0.37	0.38	0.03	ns ^2^	0.41	0.26	0.03	0.037	0.290	0.03
C14:0	1.94	2.08	0.05	ns	2.19	2.59	0.07	0.008	<0.001	0.06
C16:0	20.3	20.7	0.26	ns	24.2	25.9	0.50	ns	<0.001	0.39
C18:0	6.42	5.85	0.12	ns	7.89	6.89	0.17	0.001	<0.001	0.12
SFAs ^3^	30.9	30.8	0.24	ns	36.9	37.6	0.70	ns	<0.001	0.53
C14:1	0.02	0.10	0.01	0.011 (0.08 ^8^)	0.10	0.18	0.02	0.010 (0.08)	<0.001	0.01
C16:1	1.40	2.10	0.12	0.013 (0.70)	1.40	3.06	0.19	<0.001 (1.66)	0.004	0.11
C18:1 *n*-9	28.0	28.5	0.16	ns	25.5	28.0	0.35	<0.001	<0.001	0.22
C22:1 *n*-9	0.01	0.01	0.00	ns	0.10	0.07	0.01	0.030	<0.001	0.01
MUFAs ^4^	31.1	32.6	0.26	ns	28.9	33.2	0.55	<0.001	0.074	0.31
C18:2 *n*-6	28.8	27.7	0.29	0.889	25.5	22.1	1.03	0.107	<0.001	0.73
C18:3 *n*-6	0.10	0.10	0.01	ns	0.18	0.11	0.01	<0.001	<0.001	0.01
C20:4 *n*-6	1.85	1.74	0.07	ns	1.58	0.88	0.11	<0.001	<0.001	0.08
PUFAs ^5^	33.7	32.7	0.33	ns	29.6	25.3	1.20	ns	<0.001	0.85
UFAs ^6^/SFAs	2.11	2.12	0.02	ns	1.61	1.58	0.04	ns	<0.001	0.70
*n*-6	31.4	30.2	0.32	ns	27.5	23.3	1.12	0.045	<0.001	0.79
*n*-3	2.32	2.51	0.05	ns	2.10	2.03	0.09	ns	<0.001	0.07
*n*-6/*n*-3	13.7	12.1	0.29	0.006 (1.60)	13.4	11.5	0.28	0.002 (1.90)	0.207	0.24
PI ^7^	42.8	41.8	0.46	ns	37.9	31.3	1.53	0.017	<0.001	1.06
Identified, %	95.7	96.1			95.4	96.1				

^1^ SE: standard error; ^2^ not significant; ^3^ SFAs: saturated fatty acids; ^4^ MUFAs: monounsaturated fatty acids; ^5^ PUFAs: polyunsaturated fatty acids; ^6^ UFAs: unsaturated fatty acids; ^7^ peroxidability index: (% monoenoic × 0.025) + (% dienoic × 1) + (% trienoic × 2) + (% tetraenoic × 4) + (% pentaenoic × 6) + (% hexaenoic × 8); ^8^ difference between means.

**Table 5:** Fatty acid profile (% of total FAMEs) of the abdominal wall of Lean and Fat rabbits in third and fourth generations of divergent selection for total body fat content.

Generation (Gen)	3				4				*p*-Gen	
Line	Lean	Fat	SE ^1^	*p*-Line	Lean	Fat	SE	*p*-Line		SE
Fatty Acid Profile	% Fatty Acid				% Fatty Acid					
*n*. samples	15	15			15	15				
C16:0	20.4	20.8	0.30	ns ^2^	27.5	27.4	0.66	ns	<0.001	0.52
C17:0	0.53	0.51	0.03	ns	0.92	0.73	0.03	0.014	<0.001	0.03
C18:0	5.81	5.45	0.09	ns	8.54	7.39	0.21	0.001	<0.001	0.14
SFAs ^3^	30.5	30.6	0.34	ns	42.0	40.0	1.04	ns	<0.001	0.78
C16:1	1.54	2.39	0.14	0.012 (0.85 ^8^)	1.60	3.35	0.21	<0.001 (1.75)	0.010	0.13
C22:1 *n*-9	0.04	0.03	0.01	ns	0.15	0.09	0.01	0.020	<0.001	0.01
MUFAs ^4^	31.9	33.5	0.29	ns	32.4	35.7	0.59	0.001	0.024	0.41
C18:2 *n*-6	29.3	27.9	0.34	0.932	17.3	16.7	1.54	0.995	<0.001	1.13
C18:3 *n*-6	0.08	0.09	0.01	ns	0.19	0.14	0.01	0.019	<0.001	0.01
PUFAs ^5^	34.0	32.7	0.40	ns	20.0	19.2	1.77	ns	<0.001	1.30
UFAs ^6^/SFAs	2.17	2.17	0.03	ns	1.30	1.41	0.07	ns	<0.001	0.05
*n*-6	31.5	30.1	0.39	ns	18.7	17.7	1.64	ns	<0.001	1.20
*n*-3	2.49	2.59	0.04	ns	1.23	1.51	0.14	ns	<0.001	0.10
*n*-6/*n*-3	12.7	11.7	0.26	ns	15.9	12.5	0.49	<0.001	<0.001	0.33
PI ^7^	42.4	40.9	0.57	ns	25.8	24.2	2.12	ns	<0.001	1.58
Identified, %	96.3	96.7			94.4	94.8				

^1^ SE: standard error; ^2^ not significant; ^3^ SFAs: saturated fatty acids; ^4^ MUFAs: monounsaturated fatty acids; ^5^ PUFAs: polyunsaturated fatty acids; ^6^ UFAs: unsaturated fatty acids; ^7^ peroxidability index: (% monoenoic × 0.025) + (% dienoic × 1) + (% trienoic × 2) + (% tetraenoic × 4) + (% pentaenoic × 6) + (% hexaenoic × 8); ^8^ difference between means.

**Table 6:** Content (mg/100 g of meat) of main FA classes of the four meat portions (*longissimus thoracis et lumborum* (LTL), hind legs, forelegs, abdominal wall) of Lean and Fat rabbits in third and fourth generations of divergent selection for total body fat content.

Generation (Gen)	3			4				*p*-Gen	SE
Line	Lean	Fat	*p*-Line	Lean	Fat	*p*-Line	SE ^1^		
*n*. samples	15	15		15	15				
LTL:									
SFAs ^3^	886	991	ns ^2^	1202	1277	ns	34.2	<0.001	24.2
MUFAs ^4^	871	1063	0.014 (192 ^6^)	810	1027	0.004 (217)	42.8	ns	30.2
PUFAs ^5^	816	867	ns	1069	1020	ns	20.7	<0.001	14.7
*n*-6	768	811	ns	1015	954	ns	19.8	<0.001	14.0
*n*-3	47.8	55.5	ns	54.0	65.8	ns	3.08	0.010	2.18
Hind legs:									
SFAs	1369	1579	ns	1556	1869	0.001	56.0	<0.001	39.6
MUFAs	1488	1757	0.012 (269)	1160	1609	<0.001 (449)	58.9	<0.001	41.6
PUFAs	1378	1513	ns	1419	1479	ns	39.8	ns	28.2
*n*-6	1282	1400	ns	1329	1373	ns	35.3	ns	25.0
*n*-3	96.3	113	ns	90.0	106	ns	5.30	ns	3.74
Forelegs:									
SFAs	2602	3226	ns	2578	4014	<0.001	165	0.024	117
MUFAs	2625	3414	0.003 (789)	2015	3546	<0.001 (1531)	150	ns	106
PUFAs	2839	3385	ns	2117	2678	0.045	143	<0.001	101
*n*-6	2640	3122	ns	1974	2463	ns	130	<0.001	91.7
*n*-3	199	263	0.011 (64)	151	216	0.013 (65)	13.9	0.001	10.1
Abdominal wall:									
SFAs	2613	3540	0.014 (927)	2712	3852	0.002 (1140)	206	ns	146
MUFAs	2746	3895	<0.001 (1149)	2078	3450	<0.001 (1372)	200	0.007	141
PUFAs	2914	3727	0.023	1185	1816	ns	190	<0.001	134
*n*-6	2696	3427	0.025	1112	1673	ns	173	<0.001	122
*n*-3	218	300	0.013 (82)	73	143	0.048 (70)	18.0	<0.001	12.8

^1^ SE: standard error; ^2^ not significant; ^3^ SFAs: saturated fatty acids; ^4^ MUFAs: monounsaturated fatty acids; ^5^ PUFAs: polyunsaturated fatty acids; ^6^ difference between means.

## Data Availability

The data generated and analyzed during this study are included in this article and in the supplementary materials.

## Supplementary Materials

The following are available online at https://www.mdpi.com/article/10.3390/ani12182396/s1, Table S1: Complete fatty acid profile (% of total FAME) of the *longissimus thoracis et lumborum* of Lean and Fat rabbits in third and fourth generations of divergent selection for total body fat content., Table S2: Complete fatty acid profile (% of total FAME) of the hind legs of Lean and Fat rabbits in third and fourth generations of divergent selection for total body fat content., Table S3: Complete fatty acid profile (% of total FAME) of the fore legs of Lean and Fat rabbits in third and fourth generations of 15 divergent selection for total body fat content., Table S4: Complete fatty acid profile (% of total FAME) of the abdominal wall of Lean and Fat rabbits in third and fourth generations of divergent selection for total body fat content.
