# Supplementary material for: Rabbits Divergently Selected for Total Body Fat Content: Changes in Proximate Composition and Fatty Acids of Different Meat Portions
Source: Animals (Basel). 2022 Sep 13;12(18):2396. doi: 10.3390/ani12182396 (PMC9494993; doi:10.3390/ani12182396)
Supplement: Supplementary file 1 [file animals-12-02396-s001.zip › animals-1836322-supplementary.pdf]

animals

MDPI

Supplementary tables

Rabbits Divergently Selected for Total Body Fat Content: Changes in Proximate Composition and Fatty Acids of Different Meat Portions

Marco Cullere [1], Zsolt Szendrő [2], Zsolt Matics [3], Zsolt Gerencsér [3], Rozália Kasza [3], Tamás Donkó [3,4] and Antonella Dalle Zotte [1,*]

Table S1. Complete fatty acid profile (% of total FAME) of the *longissimus thoracis et lumborum* of Lean and Fat rabbits in third and fourth generations of divergent selection for total body fat content.

Generation	3				4				*p*-Generation	SE
Line	Lean	Fat	SE[1]	*p*-Values	Lean	Fat	SE	*p*-Values		
Fatty acid profile	% fatty acid				% fatty acid					
N. samples	15	15			15	15				
C6:0	0.00	0.00	0.00	1.000	0.17	0.12	0.02	0.173	<0.001	0.01
C8:0	0.00	0.01	0.01	0.823	0.04	0.01	0.01	0.468	0.120	0.01
C10:0	0.13	0.13	0.03	1.000	0.13	0.16	0.02	0.949	0.571	0.02
C12:0	0.07	0.05	0.02	0.977	0.07	0.10	0.02	0.857	0.466	0.02
C14:0	1.52	1.83	0.07	0.107	1.09	1.78	0.09	<0.001	0.016	0.07
C15:0	0.54	0.48	0.02	0.346	0.53	0.48	0.02	0.651	0.930	0.02
C16:0	21.3	21.6	0.30	0.908	22.8	24.1	0.20	0.040	<0.001	0.24
C17:0	0.64	0.56	0.01	0.261	0.75	0.59	0.03	0.006	0.050	0.02
C18:0	5.98	5.44	0.11	0.211	9.51	8.29	0.20	<0.001	<0.001	0.14
C20:0	0.05	0.14	0.02	0.122	0.21	0.17	0.02	0.706	0.004	0.03
C22:0	0.11	0.12	0.03	0.989	0.23	0.16	0.02	0.503	0.035	0.05
C23:0	0.32	0.29	0.07	0.980	0.00	0.00	0.00	1.000	<0.001	0.04
**SFA**	30.1	30.6	0.35	1.000	35.5	35.9	0.20	0.871	<0.001	0.29
C14:1	0.00	0.06	0.02	0.200	0.14	0.16	0.02	0.947	<0.001	0.02
C15:1	0.03	0.03	0.01	1.000	0.07	0.11	0.02	0.254	0.001	0.01
C16:1	1.73	2.81	0.17	<0.001	0.83	2.26	0.17	<0.001	<0.001	0.01
C17:1	0.04	0.14	0.03	0.051	0.20	0.27	0.01	0.286	<0.001	0.02
C18:1 *n*-9	26.7	28.1	0.43	0.308	20.5	24.1	0.49	0.000	<0.001	0.39
C18:1 *n*-11	1.37	1.49	0.06	0.625	1.57	1.40	0.05	0.319	0.457	0.04
C20:1 *n*-9	0.08	0.06	0.02	0.981	0.29	0.31	0.02	0.954	<0.001	0.30
C22:1 *n*-9	0.03	0.06	0.02	0.946	0.34	0.20	0.03	0.030	<0.001	0.03
**MUFA**	30.0	32.7	0.54	0.018	23.9	28.8	0.61	<0.001	<0.001	0.45
C18:2 *n*-6	23.6	23.0	0.24	0.677	23.8	22.8	0.32	0.267	<0.001	0.28
C18:3 *n*-6	0.02	0.02	0.01	0.999	0.15	0.17	0.01	0.844	0.763	0.01
C20:2 *n*-6	0.22	0.14	0.04	0.698	0.21	0.19	0.03	0.998	<0.001	0.04
C20:3 *n*-6	0.16	0.13	0.03	0.967	0.48	0.32	0.03	0.044	<0.001	0.03
C20:4 *n*-6	2.57	2.06	0.13	0.593	5.31	3.37	0.31	<0.001	<0.001	0.20
C22:2 *n*-6	0.00	0.00	0.00	1.000	0.10	0.09	0.02	1.000	<0.001	0.02
C18:3 *n*-3	1.57	1.69	0.04	0.737	1.10	1.47	0.08	0.009	<0.001	0.06
C20:3 *n*-3	0.05	0.01	0.01	0.961	0.22	0.24	0.05	0.989	<0.001	0.04
C20:5 *n*-3	0.03	0.03	0.01	0.990	0.22	0.14	0.02	0.007	<0.001	0.01
C22:6 *n*-3	0.00	0.00	0.00	1.000	0.05	0.01	0.01	0.197	0.0108	0.01
**PUFA**	28.3	27.1	0.34	0.330	31.6	28.8	0.43	0.001	<0.001	0.34
UFA/SFA	1.91	1.95	0.02	0.760	1.57	1.60	0.02	0.797	<0.001	0.02
n-6	26.6	25.4	0.34	0.261	30.0	26.9	0.44	0.000	<0.001	0.33
n-3	1.65	1.73	0.03	0.932	1.59	1.87	0.08	0.113	0.667	0.06
n-6/*n*-3	16.2	14.8	0.30	0.680	20.1	15.1	0.91	0.001	0.015	0.59
PI[2]	38.7	36.1	0.68	0.417	51.5	42.6	1.29	<0.001	<0.001	0.85
Identified, %	88.9	90.5			91.0	93.5				

[1]SE: Standard error; [2]Peroxidability index: (% monoenoic x 0.025) + (% dienoic x 1) + (% trienoic x 2) + (% tetraenoic x 4) + (% pentaenoic x 6) + (% hexaenoic x 8).

Table S2. Complete fatty acid profile (% of total FAME) of the hind legs of Lean and Fat rabbits in third and fourth generations of divergent selection for total body fat content.

**Generation**	**3**				**4**					
**Line**	**Lean**	**Fat**	**SE**[1]	***p*-Values**	**Lean**	**Fat**	**SE**	***p*-Values**	***p*-Generation**	**SE**
**Fatty Acid profile**	**% fatty acid**				**% fatty acid**					
N. samples	15	15			15	15				
C6:0	0.00	0.00	0.00	1.000	0.13	0.08	0.01	0.000	<0.001	0.01
C8:0	0.04	0.05	0.01	0.857	0.01	0.01	0.00	1.000	0.007	0.01
C10:0	0.33	0.33	0.02	1.000	0.28	0.22	0.02	0.493	0.006	0.02
C12:0	0.30	0.28	0.02	0.983	0.26	0.22	0.02	0.708	0.127	0.02
C14:0	1.90	2.06	0.06	0.470	1.65	2.11	0.07	0.001	0.209	0.06
C15:0	0.57	0.51	0.03	0.858	0.52	0.42	0.04	0.474	0.146	0.04
C16:0	20.4	21.2	0.33	0.533	23.1	24.5	0.26	0.101	<0.001	0.29
C17:0	0.54	0.49	0.03	0.792	0.74	0.63	0.03	0.326	<0.001	0.03
C18:0	5.87	5.56	0.11	0.524	8.47	7.59	0.14	0.001	<0.001	0.11
C20:0	0.29	0.25	0.02	0.361	0.11	0.10	0.01	0.962	<0.001	0.02
C22:0	0.13	0.12	0.02	0.971	0.10	0.08	0.01	0.828	0.153	0.02
C23:0	0.14	0.14	0.03	1.000	0.00	0.00	0.00	1.000	<0.001	0.02
**SFA**	30.5	31.0	0.40	0.900	35.1	35.9	0.24	0.869	<0.001	0.33
C14:1	0.03	0.11	0.02	0.026	0.14	0.15	0.01	0.965	0.001	0.02
C15:1	0.18	0.17	0.01	0.917	0.07	0.07	0.01	0.992	<0.001	0.01
C16:1	1.65	2.45	0.13	0.003	1.29	2.69	0.17	<0.001	0.705	0.11
C17:1	0.08	0.12	0.02	0.583	0.23	0.30	0.01	0.061	<0.001	0.02
C18:1 *n*-9	29.9	30.2	0.20	0.917	22.9	25.9	0.40	<0.001	<0.001	0.26
C18:1 *n*-11	1.23	1.34	0.05	0.539	1.25	1.30	0.03	0.957	0.891	0.04
C20:1 *n*-9	0.04	0.10	0.03	0.694	0.29	0.34	0.03	0.711	<0.001	0.03
C22:1 *n*-9	0.05	0.01	0.01	0.402	0.15	0.12	0.01	0.721	<0.001	0.01
**MUFA**	33.1	34.5	0.30	0.167	26.4	30.8	0.56	<0.001	<0.001	0.33
C18:2 *n*-6	27.0	26.0	0.34	0.528	26.7	23.8	0.43	0.001	0.015	0.34
C18:3 *n*-3	2.07	2.18	0.05	0.846	1.71	1.71	0.08	1.000	<0.001	0.06
C18:3 *n*-6	0.06	0.04	0.01	0.995	0.14	0.31	0.05	0.113	0.001	0.04
C20:2 *n*-6	0.35	0.35	0.02	1.000	0.15	0.13	0.03	0.961	<0.001	0.02
C20:3 *n*-3	0.00	0.00	0.00	1.000	0.19	0.20	0.03	0.992	<0.001	0.03
C20:3 *n*-6	0.15	0.17	0.01	0.999	0.37	0.15	0.08	0.179	0.212	0.05
C20:4 *n*-6	1.23	1.15	0.06	0.991	2.74	1.94	0.18	0.009	<0.001	0.12
C20:5 *n*-3	0.07	0.04	0.01	0.566	0.08	0.08	0.01	0.984	0.148	0.01
C22:2 *n*-6	0.00	0.00	0.00	1.000	0.14	0.10	0.02	0.666	<0.001	0.02
C22:6 *n*-3	0.00	0.00	0.00	1.000	0.05	0.02	0.01	0.008	<0.001	0.01
**PUFA**	30.9	30.0	0.40	0.656	32.3	28.5	0.53	<.001	0.941	0.40
UFA/SFA	2.11	2.09	0.03	0.962	1.66	1.65	0.02	1.000	<0.001	0.03
n-6	28.7	27.7	0.38	0.506	30.3	26.7	0.47	<.001	0.638	0.36
n-3	2.15	2.22	0.04	0.813	2.04	2.13	0.04	0.730	0.111	0.04
n-6/*n*-3	13.6	12.5	0.24	0.200	15.0	12.6	0.36	0.000	0.040	0.26
PI[2]	38.0	36.9	0.55	0.809	44.4	38.0	0.94	<.001	<0.001	0.65
Identified, %	94.5	95.4			94.1	95.2				

[1]SE: Standard error; [2]Peroxidability index: (% monoenoic x 0.025) + (% dienoic x 1) + (% trienoic x 2) + (% tetraenoic x 4) + (% pentaenoic x 6) + (% hexaenoic x 8).

Table S3. Complete fatty acid profile (% of total FAME) of the fore legs of Lean and Fat rabbits in third and fourth generations of divergent selection for total body fat content.

**Generation**	**3**				**4**					
**Line**	**Lean**	**Fat**	**SE**[1]	***p*-Values**	**Lean**	**Fat**	**SE**	***p*-Values**	***p*-Generation**	**SE**
**Fatty acid profile**	**% fatty acid**				**% fatty acid**					
N. samples	15	15			15	15				
C6:0	0.00	0.00	0.00	1.000	0.14	0.13	0.04	0.997	0.004	0.03
C8:0	0.11	0.08	0.01	0.005	0.06	0.05	0.00	0.986	<0.001	0.01
C10:0	0.52	0.45	0.03	0.520	0.44	0.34	0.02	0.197	0.008	0.03
C12:0	0.37	0.38	0.03	1.000	0.41	0.26	0.03	0.037	0.290	0.03
C14:0	1.94	2.08	0.05	0.641	2.19	2.59	0.07	0.008	<0.001	0.06
C15:0	0.49	0.52	0.03	0.973	0.58	0.46	0.05	0.403	0.860	0.04
C16:0	20.3	20.7	0.26	0.959	24.2	25.9	0.50	0.133	<0.001	0.39
C17:0	0.35	0.49	0.04	0.120	0.73	0.66	0.03	0.662	<0.001	0.03
C18:0	6.42	5.85	0.12	0.113	7.89	6.89	0.17	0.001	<0.001	0.12
C20:0	0.23	0.20	0.01	0.989	0.20	0.22	0.08	0.996	0.908	0.06
C22:0	0.09	0.10	0.01	0.973	0.07	0.05	0.01	0.816	0.030	0.01
C23:0	0.09	0.03	0.03	0.435	0.00	0.00	0.00	1.000	0.040	0.02
**SFA**	30.9	30.8	0.24	1.000	36.9	37.6	0.70	0.929	<0.001	0.53
C14:1	0.02	0.10	0.01	0.011	0.10	0.18	0.02	0.010	<0.001	0.01
C15:1	0.17	0.13	0.01	0.101	0.09	0.07	0.01	0.599	<0.001	0.01
C16:1	1.40	2.10	0.12	0.013	1.40	3.06	0.19	<0.001	0.004	0.11
C17:1	0.05	0.14	0.02	0.141	0.29	0.32	0.02	0.921	<0.001	0.02
C18:1 *n*-9	28.0	28.5	0.16	0.605	25.5	28.0	0.35	<0.001	<0.001	0.22
C18:1 *n*-11	1.39	1.32	0.04	0.917	1.05	1.14	0.06	0.834	0.002	0.06
C20:1 *n*-9	0.11	0.25	0.03	0.262	0.28	0.38	0.04	0.543	0.004	0.04
C22:1 *n*-9	0.01	0.01	0.00	1.000	0.10	0.07	0.01	0.030	<0.001	0.01
**MUFA**	31.1	32.6	0.26	0.111	28.9	33.2	0.55	<0.001	0.074	0.31
C18:2 *n*-6	28.8	27.7	0.29	0.889	25.5	22.1	1.03	0.107	<0.001	0.73
C18:3 *n*-3	2.27	2.44	0.04	0.648	1.88	1.88	0.09	1.000	<0.001	0.07
C18:3 *n*-6	0.10	0.10	0.01	0.969	0.18	0.11	0.01	<0.001	<0.001	0.01
C20:2 *n*-6	0.42	0.44	0.01	0.948	0.09	0.04	0.02	0.246	<0.001	0.01
C20:3 *n*-3	0.01	0.02	0.01	0.933	0.13	0.09	0.02	0.253	<0.001	0.01
C20:3 *n*-6	0.23	0.20	0.01	0.749	0.14	0.11	0.02	0.853	<0.001	0.02
C20:4 *n*-6	1.85	1.74	0.07	0.895	1.58	0.88	0.11	0.000	<0.001	0.08
C20:5 *n*-3	0.04	0.05	0.01	0.781	0.06	0.05	0.01	0.880	0.590	0.01
C22:2 *n*-6	0.00	0.00	0.00	1.000	0.05	0.04	0.01	0.985	<0.001	0.00
C22:6 *n*-3	0.00	0.00	0.00	1.000	0.02	0.02	0.00	0.991	<0.001	0.00
**PUFA**	33.7	32.7	0.33	0.935	29.6	25.3	1.20	0.064	<0.001	0.85
UFA/SFA	2.11	2.12	0.02	0.997	1.61	1.58	0.04	0.978	<0.001	0.70
n-6	31.4	30.2	0.32	0.873	27.5	23.3	1.12	0.045	<0.001	0.79
n-3	2.32	2.51	0.05	0.506	2.10	2.03	0.09	0.964	<0.001	0.07
n-6/*n*-3	13.7	12.1	0.29	0.006	13.4	11.5	0.28	0.002	0.207	0.24
PI[2]	42.8	41.8	0.46	0.957	37.9	31.3	1.53	0.017	<0.001	1.06
Identified, %	95.7	96.1			95.4	96.1				

[1]SE: Standard error; [2]Peroxidability index: (% monoenoic x 0.025) + (% dienoic x 1) + (% trienoic x 2) + (% tetraenoic x 4) + (% pentaenoic x 6) + (% hexaenoic x 8).

Table S4. Complete fatty acid profile (% of total FAME) of the abdominal wall of Lean and Fat rabbits in third and fourth generations of divergent selection for total body fat content.

**Generation**	**3**				**4**					
**Line**	**Lean**	**Fat**	**SE**[1]	***p*-Values**	**Lean**	**Fat**	**SE**	***p*-Values**	***p*-Generation**	**SE**
**Fatty acid profile**	**% fatty acid**				**% fatty acid**					
N. samples	15	15			15	15				
C6:0	0.00	0.00	0.00	1.000	0.47	0.33	0.07	0.464	<0.001	0.05
C8:0	0.10	0.10	0.01	0.978	0.10	0.07	0.00	0.251	0.071	0.01
C10:0	0.35	0.33	0.03	0.965	0.34	0.22	0.02	0.073	0.082	0.02
C12:0	0.35	0.31	0.02	0.774	0.32	0.21	0.03	0.093	0.057	0.02
C14:0	1.95	2.06	0.06	0.881	2.40	2.62	0.09	0.465	<0.001	0.08
C15:0	0.59	0.53	0.03	0.895	0.66	0.50	0.05	0.207	0.668	0.04
C16:0	20.4	20.8	0.30	0.970	27.5	27.4	0.66	1.000	<0.001	0.52
C17:0	0.53	0.51	0.03	0.990	0.92	0.73	0.03	0.014	<0.001	0.03
C18:0	5.81	5.45	0.09	0.595	8.54	7.39	0.21	0.001	<0.001	0.14
C20:0	0.22	0.20	0.01	1.000	0.66	0.40	0.11	0.298	0.004	0.07
C22:0	0.11	0.09	0.02	0.907	0.12	0.09	0.01	0.743	0.949	0.02
C23:0	0.11	0.14	0.03	0.858	0.00	0.00	0.00	1.000	<0.001	0.02
**SFA**	30.5	30.6	0.34	1.000	42.0	40.0	1.04	0.557	<0.001	0.78
C14:1	0.03	0.14	0.02	0.002	0.12	0.19	0.02	0.070	0.003	0.01
C15:1	0.14	0.15	0.01	0.921	0.09	0.07	0.01	0.739	<0.001	0.01
C16:1	1.54	2.39	0.14	0.012	1.60	3.35	0.21	<0.001	0.010	0.13
C17:1	0.10	0.15	0.02	0.458	0.30	0.32	0.01	0.756	<0.001	0.02
C18:1 *n*-9	28.5	29.0	0.17	0.854	28.7	29.9	0.43	0.243	0.245	0.32
C18:1 *n*-11	1.33	1.38	0.02	0.965	1.07	1.30	0.07	0.079	0.014	0.05
C20:1 *n*-9	0.20	0.27	0.04	0.847	0.40	0.46	0.05	0.909	0.003	0.05
C22:1 *n*-9	0.04	0.03	0.01	0.982	0.15	0.09	0.01	0.020	<0.001	0.01
**MUFA**	31.9	33.5	0.29	0.191	32.4	35.7	0.59	0.001	0.024	0.41
C18:2 *n*-6	29.3	27.9	0.34	0.932	17.3	16.7	1.54	0.995	<0.001	1.13
C18:3 *n*-3	2.39	2.54	0.05	0.906	1.03	1.32	0.15	0.559	<0.001	0.11
C18:3 *n*-6	0.08	0.09	0.01	0.943	0.19	0.14	0.01	0.019	<0.001	0.01
C20:2 *n*-6	0.39	0.39	0.01	1.000	0.10	0.11	0.02	0.986	<0.001	0.02
C20:3 *n*-3	0.02	0.01	0.01	0.956	0.06	0.06	0.01	0.973	<0.001	0.01
C20:3 *n*-6	0.20	0.19	0.01	0.980	0.13	0.12	0.02	0.972	0.002	0.02
C20:4 *n*-6	1.50	1.45	0.08	0.994	0.97	0.56	0.11	0.143	<0.001	0.09
C20:5 *n*-3	0.09	0.05	0.01	0.284	0.11	0.09	0.01	0.898	0.086	0.01
C22:2 *n*-6	0.00	0.00	0.00	1.000	0.05	0.03	0.01	0.125	<0.001	0.00
C22:6 *n*-3	0.00	0.00	0.00	1.000	0.03	0.04	0.01	0.753	<0.001	0.01
**PUFA**	34.0	32.7	0.40	0.958	20.0	19.2	1.77	0.991	<0.001	1.30
UFA/SFA	2.17	2.17	0.03	1.000	1.30	1.41	0.07	0.709	<0.001	0.05
n-6	31.5	30.1	0.39	0.936	18.7	17.7	1.64	0.973	<0.001	1.20
n-3	2.49	2.59	0.04	0.966	1.23	1.51	0.14	0.538	<0.001	0.10
n-6/*n*-3	12.7	11.7	0.26	0.360	15.9	12.5	0.49	<0.001	<0.001	0.33
PI[2]	42.4	40.9	0.57	0.965	25.8	24.2	2.12	0.950	<0.001	1.58
Identified, %	96.3	96.7			94.4	94.8				

[1]SE: Standard error; [2]Peroxidability index: (% monoenoic x 0.025) + (% dienoic x 1) + (% trienoic x 2) + (% tetraenoic x 4) + (% pentaenoic x 6) + (% hexaenoic x 8).
